# Quantum-mechanics-based molecular interaction fields for 3D-QSAR

**DOI:** 10.1186/1758-2946-6-S1-O10

**Published:** 2014-03-11

**Authors:** Ahmed ElKerdawy, Stefan Güssregen, Hans Matter, Matthias Hennemann, Timothy Clark

**Affiliations:** 1 Computer-Chemie-Centrum, Friedrich-Alexander-Universität Erlangen-Nürnberg, Nägelsbachstraβe 25, 91052 Erlangen, Germany; 2 Sanofi-Aventis Deutschland GmbH,R&D, LGCR, Structure, Design and Informatics, Building G 878, 65926, Frankfurt am Main, Germany; 3 Interdisciplinary Center for Molecular Materials, Friedrich-Alexander-Universität Erlangen-Nürnberg, Nӓgelsbachstraβe 49, 91052 Erlangen, Germany; 4 Centre for Molecular Design, University of Portsmouth, King Henry Building, Portsmouth PO1 2DY, UK

## 

**Figure 1:**
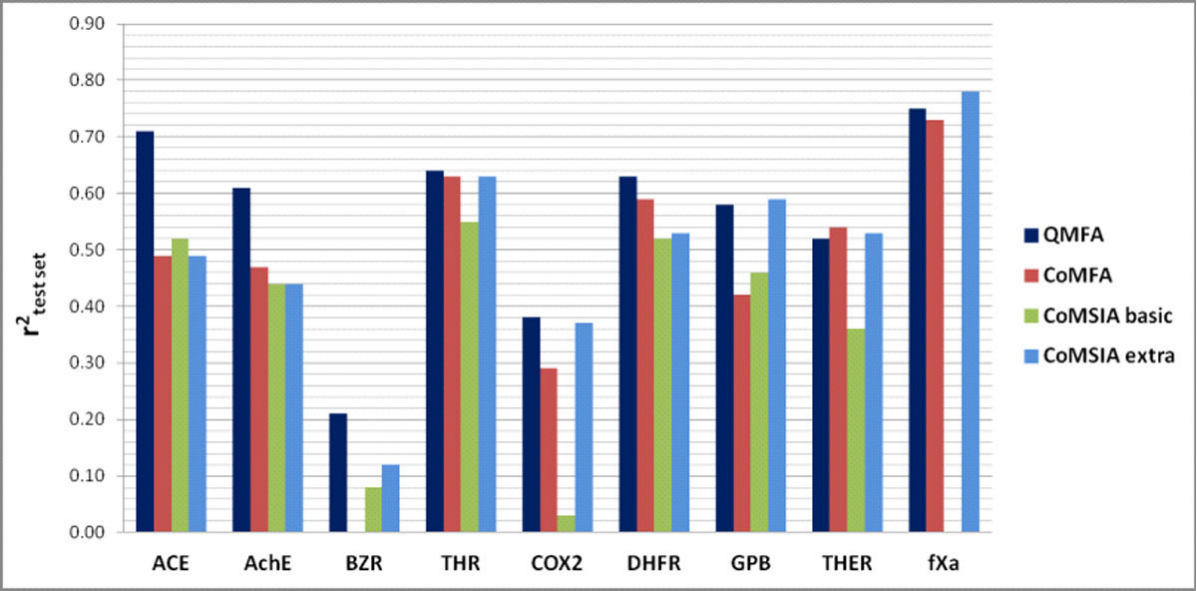
Performance of the different 3D-QSAR approaches in the external validation.

Computer-aided drug design (CADD) shift toward using quantum-mechanics (QM)-based approaches is not only the result of the ever growing computational power but also due to the need for more accurate and more informative approaches to describe molecular properties and binding characteristics than the currently available ones. QM-approaches do not suffer from the limitations inherent to the ball-and-spring description and the fixed atom-centered charge approximation in the classical force fields mostly used by CADD methods.[1] We introduce a protocol for shifting 3D-QSAR, one of the most widely used ligand-based drug design approaches, through using QM-based molecular interaction fields (MIFs) which are the electron density (ρ), hydrogen bond donor field (HDF), hydrogen bond acceptor field (HAF) and molecular lipophilicity potential (MLP) to overcome the limitations of the current force-field-based MIFs (FF-MIFs). The average performance of the QM-MIFs (QMFA) models for nine data sets was found to be better than that of the conventional FF-MIFs models. In the individual data sets, the QMFA models always perform better than, or as well as, the conventional approaches. It is particularly encouraging that the relative performance of the QMFA models improves in the external validation (Figure 1).
